# High-quantum yield alloy-typed core/shell CdSeZnS/ZnS quantum dots for bio-applications

**DOI:** 10.1186/s12951-021-01227-2

**Published:** 2022-01-06

**Authors:** Jaehi Kim, Do Won Hwang, Heung Su Jung, Kyu Wan Kim, Xuan-Hung Pham, Sang-Hun Lee, Jung Woo Byun, Wooyeon Kim, Hyung-Mo Kim, Eunil Hahm, Kyeong-min Ham, Won-Yeop Rho, Dong Soo Lee, Bong-Hyun Jun

**Affiliations:** 1 Department of Bioscience and Biotechnology, Konkuk University, Seoul, Republic of Korea; 2 Department of Nuclear Medicine, Seoul National University College of Medicine, Seoul, Republic of Korea; 3 THERABEST, Co. Inc., Seocho-daero 40-gil, Seoul, Republic of Korea; 4 Company of Global Zeus, Hwaseong, Gyeonggi-do Republic of Korea; 5 Department of Chemical and Biological Engineering, Hanbat University, Daejeon, Republic of Korea; 6 School of International Engineering and Science, Jeonbuk National University, Jeonju-si, Jeollabuk-do Republic of Korea; 7 Department of Molecular Medicine and Biopharmaceutical Sciences, Graduate School of Convergence Science and Technology and College of Medicine or College of Pharmacy, Seoul National University, Seoul, Republic of Korea; 8 KIURI Research Center, Ajou University, Suwon, Republic of Korea

**Keywords:** Quantum dot, Alloy, Surface modification, Folic acid, Quantum yield, In vitro imaging, In vivo imaging

## Abstract

**Background:**

Quantum dots (QDs) have been used as fluorophores in various imaging fields owing to their strong fluorescent intensity, high quantum yield (QY), and narrow emission bandwidth. However, the application of QDs to bio-imaging is limited because the QY of QDs decreases substantially during the surface modification step for bio-application.

**Results:**

In this study, we fabricated alloy-typed core/shell CdSeZnS/ZnS quantum dots (alloy QDs) that showed higher quantum yield and stability during the surface modification for hydrophilization compared with conventional CdSe/CdS/ZnS multilayer quantum dots (MQDs). The structure of the alloy QDs was confirmed using time-of-flight medium-energy ion scattering spectroscopy. The alloy QDs exhibited strong fluorescence and a high QY of 98.0%. After hydrophilic surface modification, the alloy QDs exhibited a QY of 84.7%, which is 1.5 times higher than that of MQDs. The QY was 77.8% after the alloy QDs were conjugated with folic acid (FA). Alloy QDs and MQDs, after conjugation with FA, were successfully used for targeting human KB cells. The alloy QDs exhibited a stronger fluorescence signal than MQD; these signals were retained in the popliteal lymph node area for 24 h.

**Conclusion:**

The alloy QDs maintained a higher QY in hydrophilization for biological applications than MQDs. And also, alloy QDs showed the potential as nanoprobes for highly sensitive bioimaging analysis.

**Graphical Abstract:**

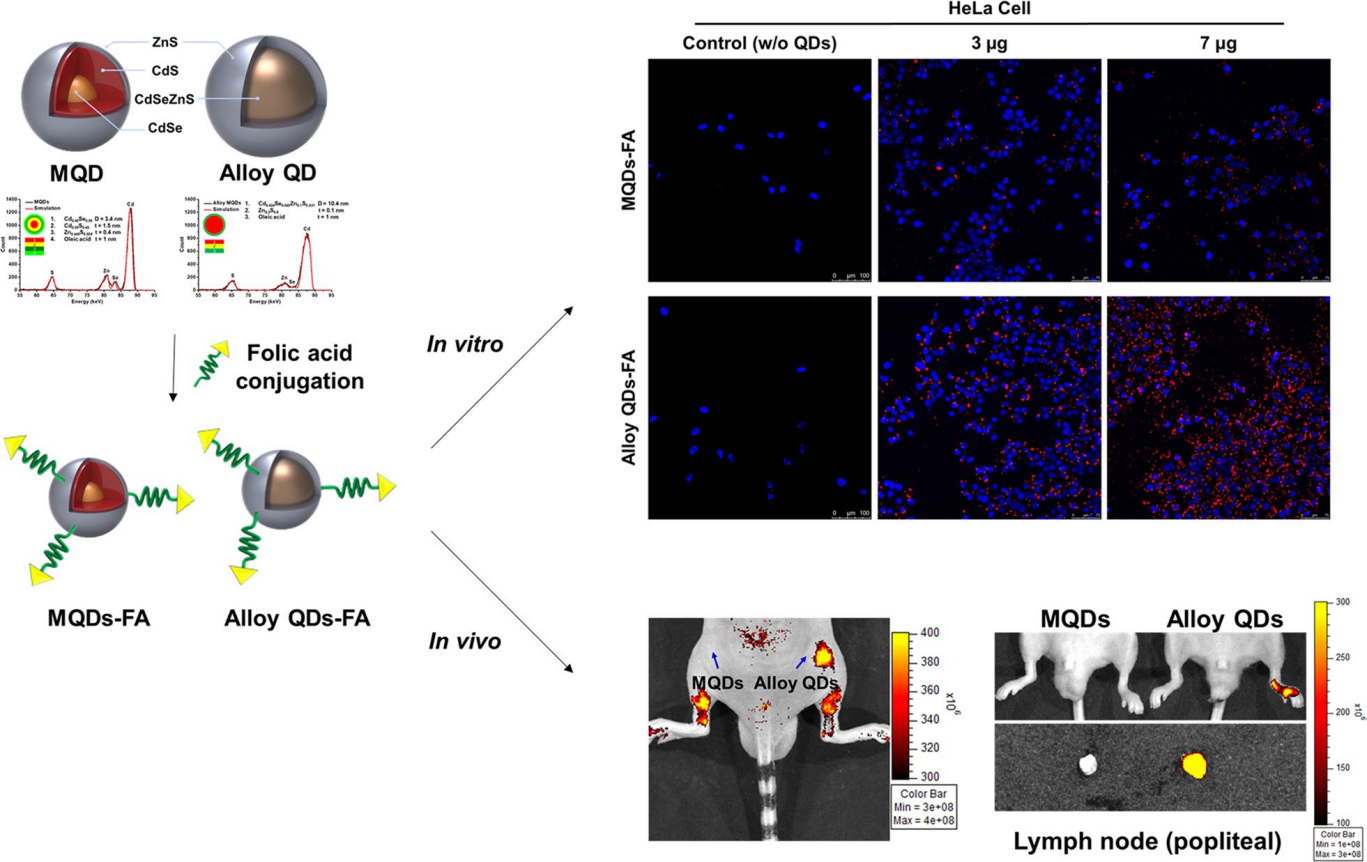

**Supplementary Information:**

The online version contains supplementary material available at 10.1186/s12951-021-01227-2.

## Background

Quantum dots (QDs) are semiconductor nanocrystals that have unique optical properties depending on their nanoscale size. QDs can absorb a wide range of light wavelengths and emit light with a narrower bandwidth than that of organic fluorophores. The maximum wavelength of the emitted light depends on the size of the QDs and can be easily adjusted by controlling the QD size. Furthermore, the photo-stability of QDs is higher than that of traditional fluorophores. Therefore, QDs have been used as an alternative to fluorophores in various fields, particularly in bio-imaging [1–4].

During QD fabrication, their surface is surrounded with ligands such as trioctylphosphine (TOP) or trioctylphosphine oxide (TOPO). Although these ligands enhance the stability of QDs in hydrophobic environments such as toluene or *n*-hexane, QDs with these ligands cannot be used for bio-imaging because they easily aggregate in such physiological conditions due to the hydrophobicity of the ligands. Therefore, several studies have attempted to modify the surface of QDs for bio-imaging applications [5–8]. However, the quantum yield (QY), an important performance index, of QDs has been reported to decrease during the surface modification step. Thus, low QY has been a major drawback of QDs, which limits their bio-application [9–11].

To address this limitation, several researchers focused on novel alloy-typed QDs instead of conventional multi-layer QDs [12–15]. Yang et al*.* reported the fabrication and application of CdSe@ZnS/ZnS core/multi-shell QDs that had an alloy-typed core [12]. The fabricated QDs had a satisfactory QY (48%) and low cytotoxicity. They could also be used for in vitro fluorescence imaging. QDs with a CdZnSeS/ZnS core–shell structure were fabricated with various thicknesses of ZnS, and their photochemical properties were analyzed [13, 14]. The QY of the fabricated QDs increased to 85% after the encapsulation of the ZnS shell, and it decreased as the thickness of the ZnS shell increased. Cho et al*.* fabricated CdSe-derived core/shell gradient alloy QDs, which had a QY of ca. 90% [15]. CdSe_x_S_1−x_/ZnSe_y_S_1−y_ alloy shells were grown gradationally onto the surface of a CdSe core, thus reducing the degree of lattice mismatch of the QDs due to the gradient conformation structure. The fabricated QDs demonstrated a high QY and exhibited photochemical stability. Although several studies have investigated alloy-typed QDs, to our best knowledge, no studies have investigated about QDs which have a high QY and their bio-imaging applications.

We previously reported on the fabrication of alloy-typed CdSeZnS/ZnS QDs (alloy QDs) with high QY and their applications for immunoassay [16]. The alloy QDs demonstrated high QY prior to antibody conjugation and exhibited high efficiency in the detection of target rabbit IgG in the immunoassay system. However, the previous study focused on only the basic properties and surface modification condition of alloy QDs; hence, it was necessary to analyze the alloy QDs in detail, including their structure and composition.

In the present study, we fabricated alloy QDs and analyzed their elemental composition for comparison with conventional CdSe/CdS/ZnS multilayer quantum dots (MQDs). Based on time-of-flight medium-energy ion scattering (TOF-MEIS) analysis method, the structures of the alloy-typed QDs and MQDs were compared. The physical properties of each type of QDs were characterized after surface modification, and both types of QDs were applied to the in vitro and in vivo bio-imaging for comparison.

## Results and discussion

### Characterization of MQDs and alloy QDs

According to previous reports, the diffusion of Zn ions into the surface of the CdSeZnS core occurs under a high reaction temperature during ZnS shell formation reaction; this phenomenon reduces the formation of structural defects and enhances the QY of the QDs [13, 14]. The gradient alloy core–shell CdSeZnS/ZnS QDs with three monolayers of ZnS shell exhibited a high QY (85%) before the surface modification, and the QY of the QDs decreased as the thickness of the outer ZnS shell decreased. Based on these previous results, we fabricated CdSeZnS/ZnS QDs with an ultrathin ZnS shell layer to enhance the QY of such QDs by more than 85%. We used additional thiol-contained ligands (1-dodecanethiol) during the fabrication of CdSeZnS core to reduce the lattice mismatch and increase the stability of the CdSeZnS core; thus, the fabricated QDs are different than existing alloy-typed QDs.

**Fig. 1:**
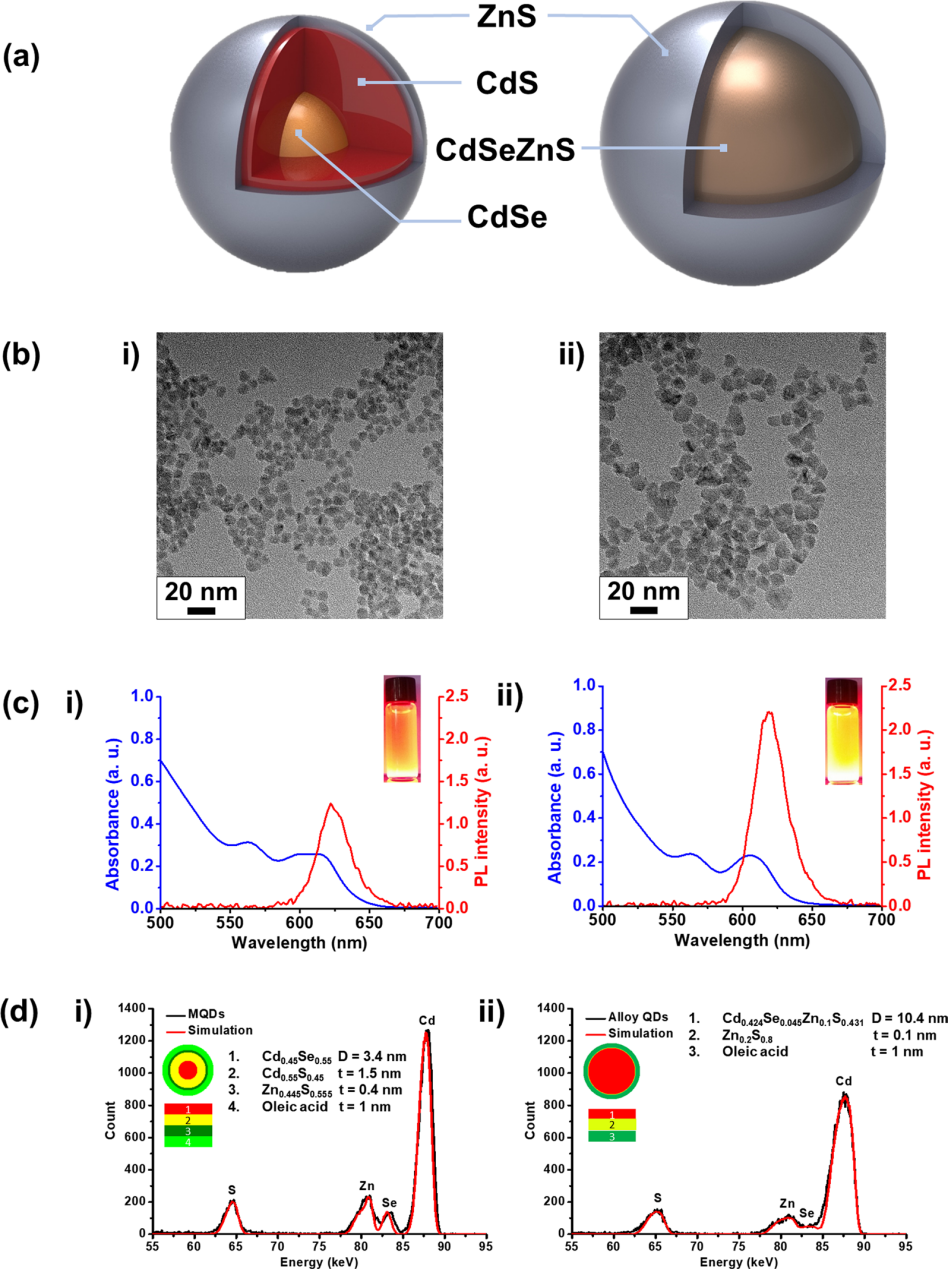
Comparison of characterizations between the conventional multi-layer quantum dot (MQD) and alloy-typed quantum dot (alloy QD). **a** Schematic representation of MQD (left) and alloy QD (right). **b** Transmission electron microscopy (TEM) images of (i) MQDs and (ii) alloy QDs. **c** UV–vis absorption spectra and PL intensities of (i) MQDs and (ii) alloy QDs (inset: fluorescence spectra under 365-nm UV light). **d** Comparison of the simulated time-of-flight-medium energy ion scattering (TOF-MEIS) spectra of (i) MQDs and (ii) alloy QDs

The intrinsic properties of the fabricated alloy QDs were compared with those of conventional MQDs. Figure 1a illustrates the structure of an MQD and an alloy QD. To confirm the size and shape, both types of QDs were analyzed using high-resolution transmission electron microscopy (Fig. 1b). The average size of the alloy QDs was 10.3 nm, with a narrow size distribution (± 2.1 nm), whereas that of the MQDs was 7.8 ± 1.7 nm, smaller than that of alloy QDs. Energy-dispersive X-ray (EDX) analysis was performed to investigate the elemental composition of the synthesized QDs (Additional file 1: Fig. S1 and Table S1). The EDX analysis revealed only Cd, Se, Zn and S; other elemental impurities were not detected, indicating that the final product was free of impurities. We also analyzed the photoluminescence (PL) and extinction spectra of the MQDs and alloy QDs (Fig. 1c). Identical quantities (1 nmol) of MQDs and alloy QDs exhibited similar UV absorption spectra patterns, but the PL intensity of alloy QDs was about twice as high as that of MQDs.

We used a TOF-MEIS system to accurately identify the structure of the MQDs and alloy QDs [17]. Because the collection efficiency of TOF-MEIS system is three orders of magnitude higher than that of conventional MEIS, the information regarding the crystallinity of specimens can be obtained, and, consequently, the composition of ultra-thin surfaces and interfaces at the nanometer level can be determined. Thus, we could analyze the composition, diameter, and the thickness of each QD. Furthermore, the number of conjugated perfluorooctanethiol (PFOT) molecules per QD was determined using the TOF-MEIS system. Figure 1d shows the TOF-MEIS spectrum of each QD analyzed using the Power-MEIS program. The simulated core–shell and conjugated layer structure of a QD along with the composition and thickness information were obtained as shown in this figure. The TOF-MEIS results indicated that each QD has a well-defined core–shell structure without significant inter-diffusion between the core and the shell, confirming that the core of alloy QDs exists in was an alloy. According to the results, MQDs had a CdSe core with a diameter of 3.4 nm as well as layers of CdS, ZnS, and oleic acid with thicknesses of 1.5 nm, 0.4 nm, and 1 nm, respectively. In contrast, alloy QDs consisted of a CdSeZnS alloy core with a diameter of 10.4 nm as well as a ZnS shell layer with a thickness of 0.1 nm and an oleic acid layer with a thickness of 1 nm. The thickness and conformation of each layer was confirmed directly with the TOF-MEIS method in this study; in contrast, only the thickness of the outer shells could be calculated indirectly from the size change of QDs via transmission electron microscopy (TEM) images in previous studies. Moreover, the TOF-MEIS method enabled the examination of thin layers that had thicknesses less than 1 nm. These TOF-MEIS results were also confirmed by TEM–EDX analysis (Additional file 1: Fig. S2).

### Surface modifications of MQDs and alloy QDs

**Fig. 2:**
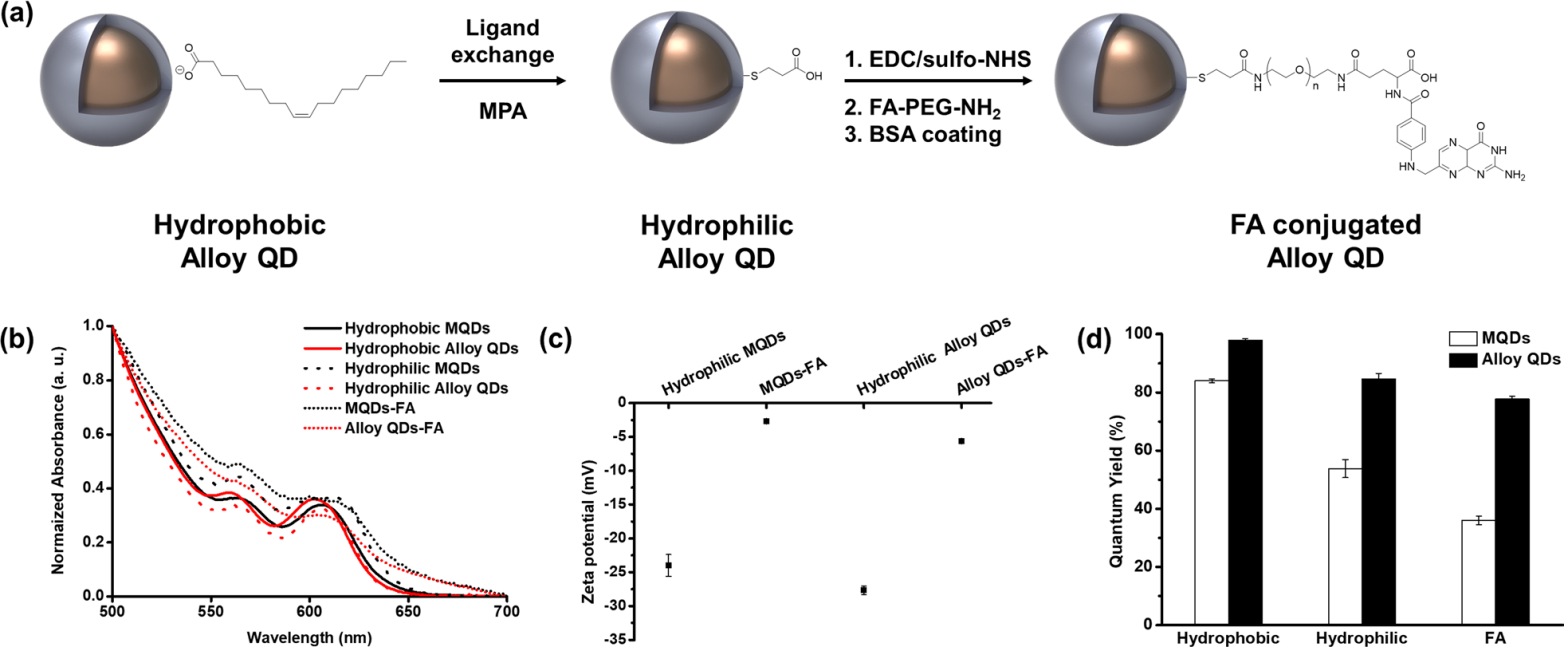
**a** Illustrated scheme for the surface modification of alloy QD. **b** Comparison of UV–Vis absorption spectra of MQDs and alloy QDs at each step. **c** Change in the zeta potential of each QD after the conjugation of FA. **d** Comparison of the QYs of the MQDs and alloy QDs at each step

To enhance the hydrophilicity and biocompatibility of MQDs and alloy QDs, we modified their surfaces (Fig. 2a). The ligand on the surface of each QD was replaced with 3-mercaptopropionic acid (MPA) to introduce a carboxyl group, which has hydrophilicity due to its negative charge. Subsequently, folic acid-conjugated MQDs (MQDs-FA) and alloy QDs (alloy QDs-FA) were fabricated with the coupling of folic acid PEG amine (FA-PEG-NH_2_) via the 1-ethyl-3-(3-dimethylaminopropyl)-carbodiimide (EDC)/*N*-hydroxysulfosuccinimide (sulfo-NHS) coupling method. Folic acid which conjugated with QDs can bind with folate receptors located on the surface of a HeLa cell or human KB cell, and furthermore, folic acid conjugated particles can be internalized into the cells via receptor-mediated endocytosis [18]. With these reason, folic acid conjugated QDs (MQDs-FA and alloy QDs-FA) can be used as fluorescence probes for imaging these cells [19, 20]. Both types of QDs were coated with bovine serum albumin (BSA) to prevent their non-specific binding during bio-imaging. The TEM images of MQDs-FA and alloy QDs-FA confirmed that the morphology and elemental positioning of the QDs after surface modification did not change significantly compared with the QDs before the surface modification (Fig. 1b, Additional file 1: Figs. S3, S4).

To examine the influence of surface modification on the physical properties of QDs, we obtained the UV–vis absorption spectra of the QDs in each step (Fig. 2b). The maximum absorption wavelengths of hydrophobic MQDs, hydrophobic alloy QDs, hydrophilic MQDs, hydrophilic alloy QDs, MQDs-FA, and alloy QDs-FA were 605, 602, 607, 605, 599, and 604 nm, respectively. There was no significant difference in the UV–vis absorption spectra between the QDs. In addition, the maximum absorption wavelengths of MQDs-FA and alloy QDs-FA are consistent with those of previous studies [21].

We also studied the change in the zeta potential of the QDs after the conjugation of FA (Fig. 2c). The zeta potentials of hydrophilic MQDs, MQDs-FA, hydrophilic alloy QDs, and alloy QDs-FA in water were − 24.0 ± 1.6, − 2.7 ± 0.1, − 27.7 ± 0.6, and − 5.6 ± 0.3 mV, respectively. Usually, carboxylic acid groups introduced onto the surface of the QDs before conjugation exist in the carboxylate form in water; thus, the zeta potential of the QDs is negative. Meanwhile, the negative zeta potentials of the QDs decreased after the conjugation of FA because the number of carboxylic acid groups decreases due to the conjugation of FA with the carboxylic acid group.

The QY of the fabricated QDs was measured and compared with other QDs (Fig. 2d). The QY of alloy QDs was 98.0 ± 0.6%, which was much higher than that of MQDs (84.0 ± 0.7%) and previously reported alloy-typed CdZnSeS/ZnS QDs (85%) [13]. According to the previous reports, the QY of alloy-typed CdZnSeS/ZnS QDs increases as the thickness of the ZnS shell decreases. Alloy-typed CdZnSeS/ZnS QDs with 85% of QY had a ZnS shell with a thickness of approximately 1.3 nm, whereas the alloy QDs fabricated in this study had a thickness of approximately 0.1 nm. Therefore, it was reasonable that our alloy QDs exhibited a higher QY than that of previously reported CdZnSeS/ZnS QDs. Next, the QY of alloy QDs was measured during surface modification and compared with that of MQDs to prove the photo-stability of the alloy QDs during surface modification. As shown in Fig. 2d, the QY of each QDs was decreased during surface modification, due to the treatment of harsh chemicals such as ammonium hydroxide during surface modification. These chemicals gave damage to the ligands and QDs, so the degree of lattice mismatch in QDs could be increased and QY of QDs might be decreased, as results. Nevertheless, the QY of alloy QDs was much higher than that of MQDs after hydrophilic modification (84.7 ± 1.8% vs. 56.8 ± 3.1%) and FA-PEG-NH_2_ conjugation (77.8 ± 0.9% vs. 36.1 ± 1.5%). With regard to the decrease rate of QY, the QY of alloy QDs decreased by approximately 20% during surface modification, whereas that of MQDs decreased by more than 50%. FA-PEG-NH_2_ as well as 3-azido-1-propanamine and dopamine hydrochloride, which contain amine groups, were conjugated with QDs for comparison. The QY of alloy QDs after conjugation was almost the same as that of QDs conjugated with FA-PEG-NH_2_ QDs (Additional file 1: Fig. S5). These results revealed the potential of alloy QDs for bio-application and indicated their QY retentivity during surface modification and bio-conjugation. This feature is comparable to that of other QD-based probes. For comparison purposes, we measured the QY of Qdot™ 625 ITK™ Carboxyl Quantum Dots in Invitrogen™ before and after the conjugation of FA-PEG-NH_2_ (Additional file 1: Fig. S6). The QY of these QDs was 75.4 ± 3.7% before conjugation; however, it considerably decreased to 54.0 ± 1.2% (decrease of up to 28%) after conjugation. This rate of decrease was higher than that of alloy QDs. The QY measurement results revealed that alloy QDs were more stable and superior than commercially available QDs during the conjugation of biomolecules such as folic acid.

### In vitro cancer specific binding study of alloy QDs-FA

**Fig. 3:**
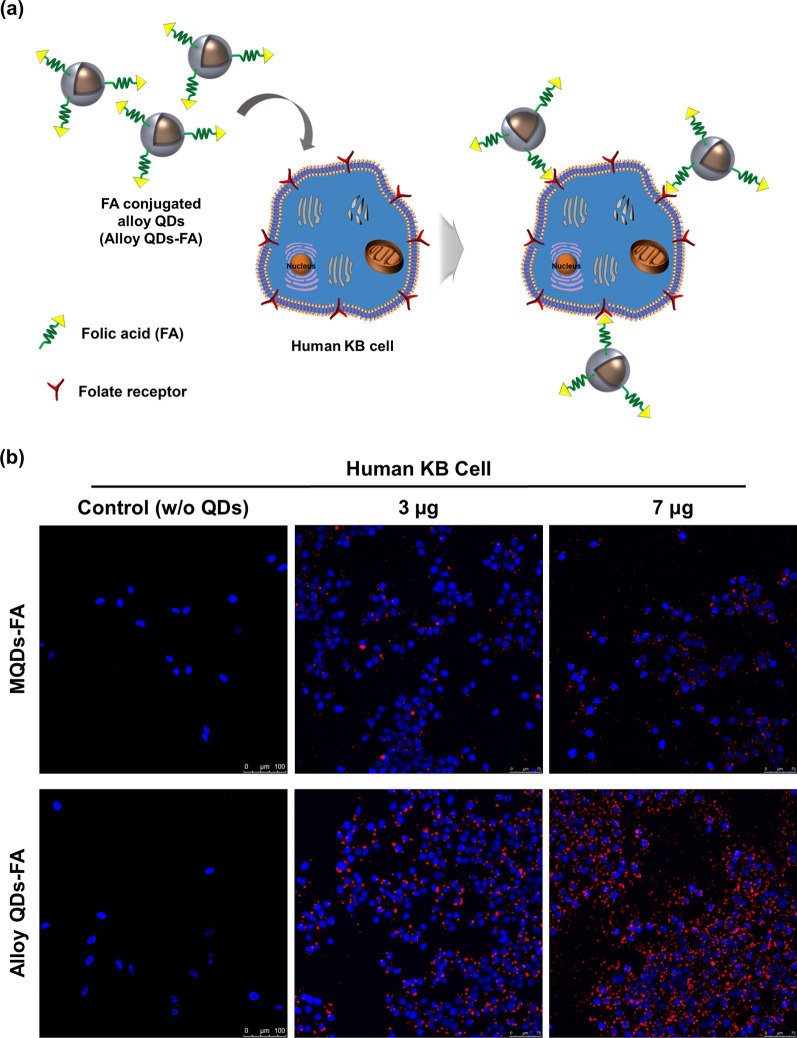
In vitro cancer targeting with folic-acid-conjugated alloy QDs (alloy QDs-FA). **a** Schematic illustration of the interaction between alloy QDs-FA and human KB cells. **b** Fluorescence image of human KB cells with folic-acid-conjugated QDs (blue: nucleus of human KB cells; red: QDs). MQDs-FA and alloy QDs-FA were treated into human KB cells in a dose-dependent manner

Next, we investigated the bio-applicability of the fabricated alloy QDs to determine whether the cancer-specific ligand-loaded alloy QDs had a cancer-specific binding ability in vitro. As a prior test, cytotoxicity of fabricated QDs (MQDs, MQDs-FA, alloy QDs, and alloy QDs-FA) were conducted with CCK8-assay, and it was confirmed that fabricated QDs were not shown the cytotoxicity in the identical amounts (3 or 7 µg, Additional file 1: Fig S7). Corresponding amounts (3 or 7 µg**)** of alloy QDs-FA and MQDs-FA were added into human KB cells, which are known to express a high level of folate receptor α (FR), for comparison (Fig. 3a) [22, 23]. As shown in Fig. 3b, QD fluorescence signals were clearly detected in FR-positive KB cells and distributed in the cell membrane area in the confocal image when treated with alloy QDs-FA. Fluorescence signals of MQDs-FA were not detected as clearly. Furthermore, a dose-dependent increment in fluorescence intensity was clearly observed in cells treated with alloy QDs-FA as compared with those treated with MQDs-FA.

### In vivo fluorescence imaging of highly sensitive alloy QDs

**Fig. 4:**
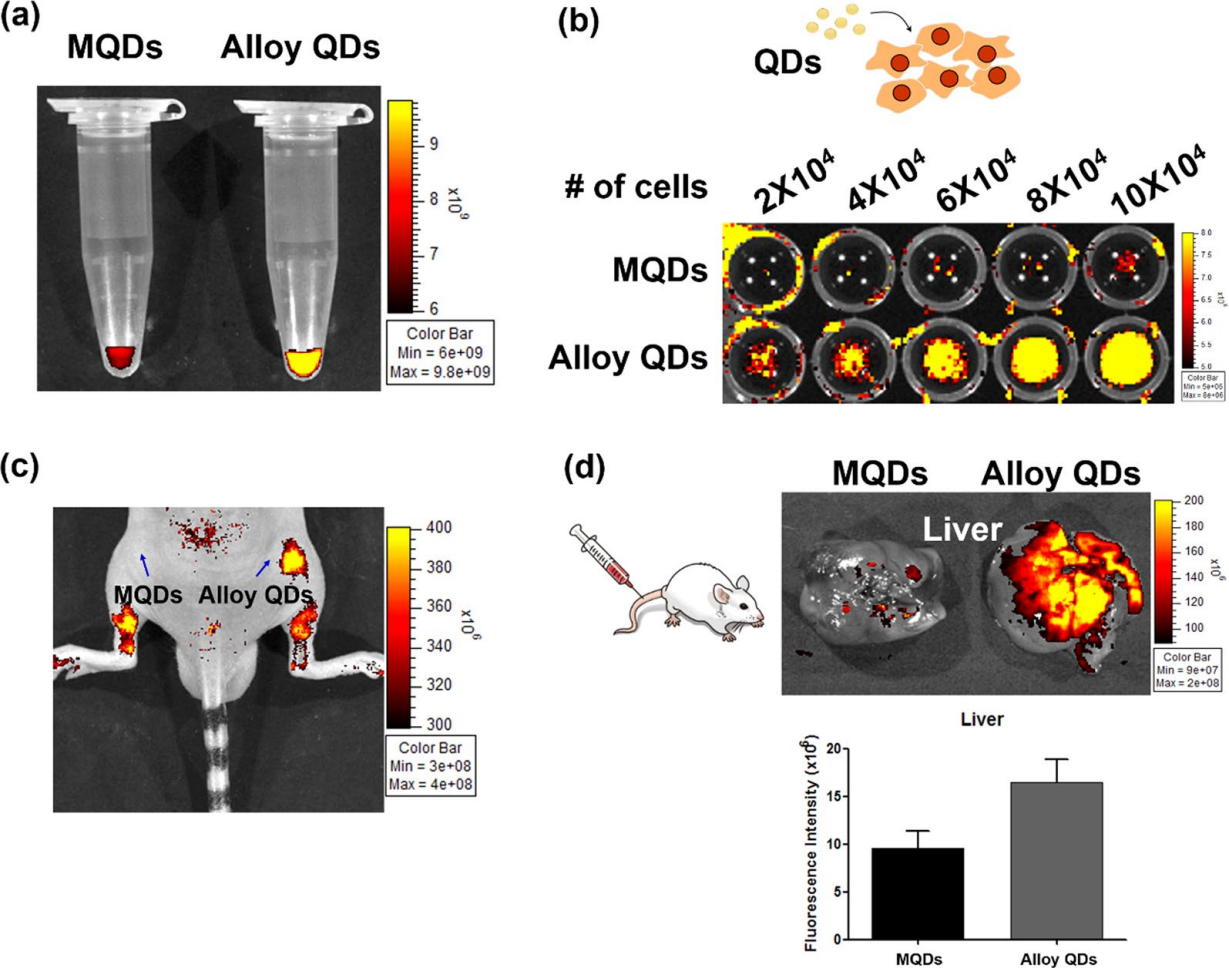
In vivo fluorescence imaging of sensitive alloy QDs. **a** Measured fluorescence intensity of the same amount of MQDs and alloy QDs measured in a tube using an in vivo imaging device. Higher fluorescence signal from alloy QDs than that of MQDs seen in the tube. **b** MQDs or alloy QDs were internalized into HeLa cells, and a cell-number-dependent increase in the fluorescence signal was detected in the alloy QDs treated group. **c** The same concentration of MQDs and alloy QDs were subcutaneously injected into nude mice. **d** Fluorescence image obtained 1 h after the intravenous injection of MQDs or alloy QDs. The region of interest (ROI) was drawn in the liver tissue of each group

To examine in vivo sensitivity of alloy QDs, we first measured the fluorescence signals of alloy QDs in a microtube. Identical amounts of MQDs and alloy QDs were transferred into the microtube, and fluorescence images were obtained using an in vivo imaging device. The fluorescence signals of alloy QDs were more intense those of MQDs in the microtube (Fig. 4a). Furthermore, the fluorescence signals increased as the number of cells increased in the alloy QDs-treated group, but the increase was not observed clearly in the MQDs-treated group (Fig. 4b). Four hours after the HeLa cells were incubated with either MQDs or alloy QDs, the cells were implanted into a nude mouse subcutaneously (Fig. 4c). The sites where alloy QDs-treated HeLa cells were injected showed high fluorescence intensity. However, there was weak fluorescence intensity at the site where MQDs-treated HeLa cells were injected.

Generally, intravenously administered nanomaterials with sizes ranging from 10 to 500 nm show a high accumulation rate in the liver tissue [24–26]. To compare the fluorescence intensities of the two different QD types when retained in the liver, identical amounts of MQDs and alloy QDs were administered into each mouse intravenously (Fig. 4d). High fluorescence signals were found to be distributed in the liver area after treatment with alloy QDs. Even though the liver of alloy QDs injected mice showed high fluorescence intensity, no noticeable cytotoxicity and hepatoxicity was observed (see Additional file 1: Table S2, Fig S8). These results demonstrated that the fabricated highly sensitive alloy QDs were more suitable for bio-imaging than MQDs, which was also verified after a semi-quantitative analysis of fluorescence intensity in the liver tissue by drawing the region of interest.

### In vivo lymph node mapping using alloy QDs

**Fig. 5:**
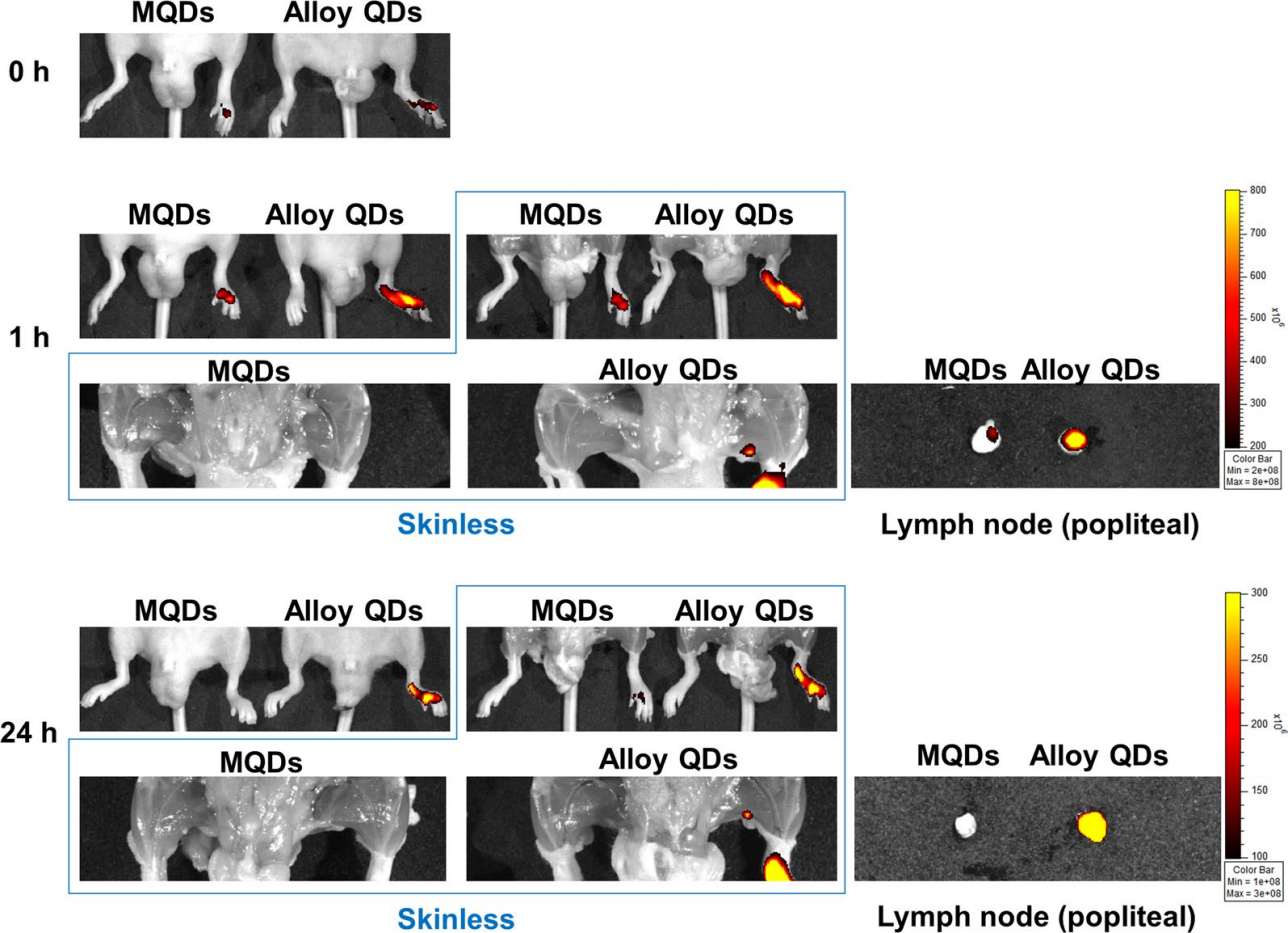
Lymph node mapping in mice injected with alloy QDs. MQDs or alloy QDs were injected into the footpad of the mice. The fluorescence signal in the injected QDs was observed in the injection site, and the injected QDs were delivered to lymph node site via lymphatic vessels. Fluorescence signals were observed only in the alloy-QD-injected group 1 h and 24 h after injection. Fluorescence signal in alloy QDs was markedly observed in the popliteal lymph node

Examining the location of the lymph node rapidly and precisely is crucial for investigating cancer progression or cancer therapy [27]. The development of techniques for lymph node mapping based on sensitive and imageable nanomaterials can offer beneficial information related to the image-guided dissection of lymph node with greater precision [28–30]. If antigen-loaded nanomaterials are developed as a vaccine for cancer, such sensitive nanomaterials may help understand the amount or the retention time of the nanovaccine in primary or secondary lymph nodes [31]. To verify whether the highly sensitive alloy QDs could trace the lymph node, we injected alloy QDs and MQDs separately into the footpad of each mouse for lymph node mapping (Fig. 5). The injected QDs moved to lymph node via the lymphatic vessel. One hour after injection, fluorescence signals were clearly observed at the popliteal lymph nodes only in the alloy QDs-injected group. When popliteal lymph nodes were isolated, the fluorescence signals in the alloy QDs-treated lymph node were stronger than those in the MQDs-treated lymph node. Fluorescence signals were still observed in the lymphatic vessel 24 h after injection, and the fluorescence signals of the alloy QDs remained in the popliteal lymph node area. With these results, it was proved that alloy QDs were adoptable to in vivo imaging as fluorescence probe, with their high QY compared with MQDs.

## Conclusions

We fabricated high-quality alloy-typed CdSeZnS/ZnS QDs (alloy QDs) and compared their properties with those of conventional multi-layer CdSe/CdS/ZnS QDs (MQDs) for evaluation. The exact structure of alloy QDs was confirmed with TOF-MEIS. These QDs exhibited stronger fluorescence signals than those of MQDs. The fabricated hydrophobic alloy QDs exhibited a QY of 98.0 ± 0.6%, which was higher than that of the MQDs (84.0 ± 0.7%). After ligand exchange, the alloy QDs exhibit a QY of 84.7 ± 1.8%, which was not significantly less, whereas the MQDs exhibited a QY of 56.8 ± 3.1%, which considerably less than that prior to surface modification. After FA conjugation, alloy QDs-FA exhibit a QY of 77.8 ± 0.9% of QY, which was similar to that (79.4%) before surface modification. In experiments targeting human KB cells, each QD type yielded different results. The alloy QDs-FA-treated group was superior than MQDs-FA-treated group. When QD-treated HeLa cells were implanted into nude mice subcutaneously, intense fluorescence images from subcutaneously injected alloy QDs were markedly observed. When the QDs were intravenously administered, the fluorescence signals from the alloy QD-injected group were stronger than those of the MQD-injected group. In a lymph node tracking system using the QDs, fluorescence signals from alloy QDs were still observed along with lymphatic vessel and remained in the popliteal lymph node area until 24 h after injection. Therefore, fabricated alloy QDs are expected to be useful for bio-imaging applications including cell tracking, which requires high sensitivity.

## Methods

### Materials

All reagents were used as received from the suppliers without further purification. Cadmium oxide (CdO, 99.9%) was purchased from Alfa Aesar (Ward Hill, MA, USA). Tri-*n*-octylphosphine (TOP, 97%) was purchased from STREM Chemicals (Newburyport, MA, USA). Selenium (Se, 99.5%) was purchased from Acros Organics (Geel, Belgium). Sulfur (S, 99%) and ammonium hydroxide (NH_4_OH, 27%) were purchased from Daejung (Siheung, Korea). Folic acid PEG amine (FA-PEG-NH_2_) was purchased from Nanocs Inc. (New York, NY, USA). Zinc acetate (Zn(OAc)_2_, 99.99%), oleic acid (OA, 90%), 1-octadecene (ODE, 90%), 1-octanethiol (98.5%), acetone (99.9%), 3-mercaptopropionic acid (MPA, 99%), 1-dodecanthiol (98%), chloroform (CHCl_3_, 99.5%), 1-ethyl-3-(3-dimethylaminopropyl)carbodiimide (EDC) hydrochloride, *N*-hydroxysulfosuccinimide (sulfo-NHS), phosphate buffered saline (PBS, pH 7.4), TWEEN® 20, and bovine serum albumin (BSA) were purchased from Sigma Aldrich (St. Louis, MO, USA). KB cell lines were purchased from American Type Culture Collection (ATCC, Manassas, VA, USA). Fetal bovine serum and Roswell Park Memorial institute (RPMI) medium were purchased from Welgene (Daegu, Korea). Antibiotics (penicillin and streptomycin) were purchased from Gibco (Grand Island, NY, USA). Mounting medium containing 4′,6-diamidino-2-phenylindole (DAPI) was purchased from Vectashield (Burlingame, CA, USA).

### Preparation of MQDs

The hydrophobic MQDs were prepared by according to the following method. First, 75 mL of ODE, 15 mL of OA, 1.1 g of Zn(OAc)_2_, and 0.384 g of CdO were put into a three-necked flask which dried in vacuum at 150 °C for 1 h. The mixture was heated to 300 °C in the presence of nitrogen gas. Next, 0.6 mL of TOP and 0.048 g of Se were added to the mixture for 3 min. Subsequently, 0.5 mL of 1-dodecanthiol was added to the mixture, and the contents were allowed to react for 20 min; followed by adding 3 mL of TOP and 0.192 g of S and allowing the contents to react for 10 min. Next, 1 mL of 1-dodecanthiol was added, and the contents were reacted for 10 min, followed by cooling at room temperature. Prepared MQDs were purified via precipitation with acetone, and collected MQDs were dispersed in CHCl_3_.

### Preparation of alloy QDs

The hydrophobic alloy QDs were prepared by according to the following method. First, 60 mL of ODE, 10 mL of OA, 1.1 g of Zn(OAc)_2_, and 0.384 g of CdO were put into a three-necked flask which dried in vacuum at 150 °C for 1 h. The mixture was heated to 300 °C in the presence of nitrogen gas. The prepared MQDs were added to the mixture, and the contents were allowed to react for 10 min. Subsequently, 3 mL of TOP and 0.192 g of S were added to the mixture, and the contents were allowed to react for 10 min, followed by cooling at room temperature. Prepared alloy QDs were purified via precipitation with acetone, and collected alloy QDs were dispersed in CHCl_3_.

### Ligand exchange of QDs

The ligand of each QDs was exchanged according to a previously reported procedure with some modification [16]. First, the reaction solution was prepared with 1.0 mL of MPA, 1 mL of NH_4_OH, and 30 mL of CHCl_3_ in a falcon tube. The reaction mixture was mixed in a rotating shaker for 2 h. Subsequently, 10 mg of each QDs, 10 mL of distilled water, and 10 mL of reaction solution were mixed in the falcon tube and reacted in a rotating shaker for 2 h. The mixture was centrifuged, and the supernatant was decanted. The residual QDs were washed with CHCl_3_ several times. After washing with acetone, the QDs were purified with an Amicon filter and dispersed in distilled water.

### Fabrication of folic acid conjugated MQDs (MQDs-FA) and alloy QDs (alloy QDs-FA)

For conjugation of folic acid onto each QD type, the QDs were incubated with sulfo-NHS (2 mg) and EDC hydrochloride (2 mg), and reacted for 2 h. at room temperature. The reaction mixture was washed several times with PBS buffer solution at 4 °C. FA-PEG-NH_2_ was added to the NHS-activated each QDs (in 200 µL PBS buffer solution) and reacted for 1 h at room temperature. The reaction mixture was washed with PBS buffer solution containing 0.1 wt% TWEEN® 20. Subsequently, the QDs were re-dispersed in 5% BSA and reacted in a vortex for 1 h. The reaction mixture was washed with PBS buffer solution containing 0.1 wt% TWEEN® 20, and the folic acid conjugated QDs were dispersed in PBS (pH 7.4).

### Characterization of the particles

The TEM images of QDs were obtained using Carl Zeiss LIBRA 120 (Oberkochen, Germany). The extinction properties of QDs were analyzed using a UV–vis spectrophotometer (Mecasys OPTIZEN POP, Daejeon, Korea). The photoluminescence intensities were obtained using a fluorescence spectrophotometer (Model Cary Eclipse, Agilent Technologies, Santa Clara, CA, USA). The morphology and surface of the QDs were analyzed using a TOF-MEIS system (MEIS-K120; K-MAC, Korea). The quantum yield was measured using a quantum efficiency measurement System (QE-2000; Otsuka Electronics, Japan).

### In vitro cell binding test using MQDs-FA and alloy QDs-FA

KB cell line, the human nasopharyngeal cancer cell line, was cultured using a folate-deficient Roswell Park Memorial Institute (RPMI) medium with 10% FBS and 1% antibiotics (penicillin and streptomycin). Subsequently, 5 × 10^5^ of KB cells were seeded into a round cover slip on a 24-well plate. Twenty-four hours after KB cell seeding, 3 or 7 µg of the MQDs-FA or alloy QDs-FA were added into the KB cells. The 24-well plates were incubated at 4 °C in the presence of 5% CO_2_ for 30 min. Subsequently, the KB cells were mounted in an aqueous mounting medium containing DAPI solution.

### In vivo fluorescence imaging of alloy QDs for bio-imaging

For the subcutaneous injection study, HeLa cells were incubated with either MQDs or alloy QDs; after 4 h, the cells were detached and implanted into the subcutaneous area of BALB/c nude mice. For lymph node imaging, 7-week-old BALB/c male mice were anesthetized using isoflurane; identical amounts of MQDs or alloy QDs were injected into the left hind footpad area of the mice. Fluorescence images were acquired at 0, 1, and 24 h after injection using an IVIS-100 imaging device, at which time the popliteal lymph node of the mice was resected.

## Supplementary Information

**Additional file 1.** EDX spectrum and atomic composition analysis of MQDs and alloy QDs; TEM–EDX images of MQDs and alloy QDs; TEM images of MQDs-FA and alloy QDs-FA; TEM–EDX images of MQDs, alloy QDs, MQDs-FA, and alloy QDs-FA; QY of hydrophobic MQDs/alloy QDs, hydrophilic MQDs/alloy QDs, 3-azido-1-propanamine conjugated MQDs/alloy QDs, and dopamine conjugated MQDs/alloy QDs; QY of Qdot™ 625 ITK™ Carboxyl Quantum Dots in Invitrogen™ before and after conjugation of FA; Cell viability test of fabricated QDs via CCK-9 assay; Protein levels of mice after 24 h from injection of PBS or QDs; Liver hematoxylin and eosin (H&E) staining of PBS, MQDs, and alloy QDs treated mice; Immunohistochemistry staining for caspase-3 detection of PBS, MQDs, and alloy QDs treated mice; Caspase-3 level of PBS, MQDs, and alloy QDs treated mice.

## Data Availability

All data generated or analyzed during this study are included in this manuscript and its Additional file.

## Abbreviations

QDs: Quantum dots
QY: Quantum yield
alloy QDs: Alloy typed core/shell quantum dots
MQDs: Multi-layer quantum dots
TOF-MEIS: Time-of-flight medium-energy ion scattering spectroscopy
FA: Folic acid
TOP: Trioctylphosphine
TOPO: Trioctylphosphine oxide
PMAH: Polymaleic acid *n*-hexadecanol ester
FA-PEG-NH_2_: Folic acid PEG amine
OA: Oleic acid
ODE: 1-Octadecene
MPA: 3-Mercaptopropionic acid
FBS: Fetal bovine serum
EDC: 1-Ethyl-3-(3-dimethylaminopropyl)carbodiimide
sulfo-NHS: *N*-Hydroxysulfosuccinimide
PBS: Phosphate buffered saline
BSA: Bovine serum albumin
DAPI: 4′,6-Diamidino-2-phenylindole
MQDs-FA: Folic acid conjugated MQDs
alloy QDs-FA: Folic acid conjugated alloy QDs
TEM: Transmission electron microscopy
UV–Vis: Ultraviolet–visible light
EDX: Energy dispersive spectroscopy
