# Supplementary material for: High-quantum yield alloy-typed core/shell CdSeZnS/ZnS quantum dots for bio-applications
Source: J Nanobiotechnology. 2022 Jan 6;20:22. doi: 10.1186/s12951-021-01227-2 (PMC8739727; doi:10.1186/s12951-021-01227-2)
Supplement: Supplementary file 1 — Additional file 1. EDX spectrum and atomic composition analysis of MQDs and alloy QDs; TEM–EDX images of MQDs and alloy QDs; TEM images of MQDs-FA and alloy QDs-FA; TEM–EDX images of MQDs, alloy QDs, MQDs-FA, and alloy QDs-FA; QY of hydrophobic MQDs/alloy QDs, hydrophilic MQDs/alloy QDs, 3-azido-1-propanamine conjugated MQDs/alloy QDs, and dopamine conjugated MQDs/alloy QDs; QY of Qdot™ 625 ITK™ Carboxyl Quantum Dots in Invitrogen™ before and after conjugation of FA; Cell viability test of fabricated QDs via CCK-9 assay; Protein levels of mice after 24 h from injection of PBS or QDs; Liver hematoxylin and eosin (H&E) staining of PBS, MQDs, and alloy QDs treated mice; Immunohistochemistry staining for caspase-3 detection of PBS, MQDs, and alloy QDs treated mice; Caspase-3 level of PBS, MQDs, and alloy QDs treated mice. [file 12951_2021_1227_MOESM1_ESM.docx]

**Additional file 1**

**High Quantum Yield Alloy Typed Core/shell CdSeZnS/ZnS Quantum Dots for Bio-applications**

Jaehi Kim,^1+^ Do Won Hwang,^2,3+^ Heung Su Jung,^4+^ Kyu Wan Kim,^2^ Xuan-Hung Pham,^1^ Sang-Hun Lee,^5^ Jung Woo Byun,^2^ Wooyeon Kim,^1^ Hyung-Mo Kim,^1^ Eunil Hahm,^1^ Kyeongmin Ham,^1^ Won-Yeop Rho,^6^ Dong Soo Lee^2,7^* and Bong-Hyun Jun^1^*

**
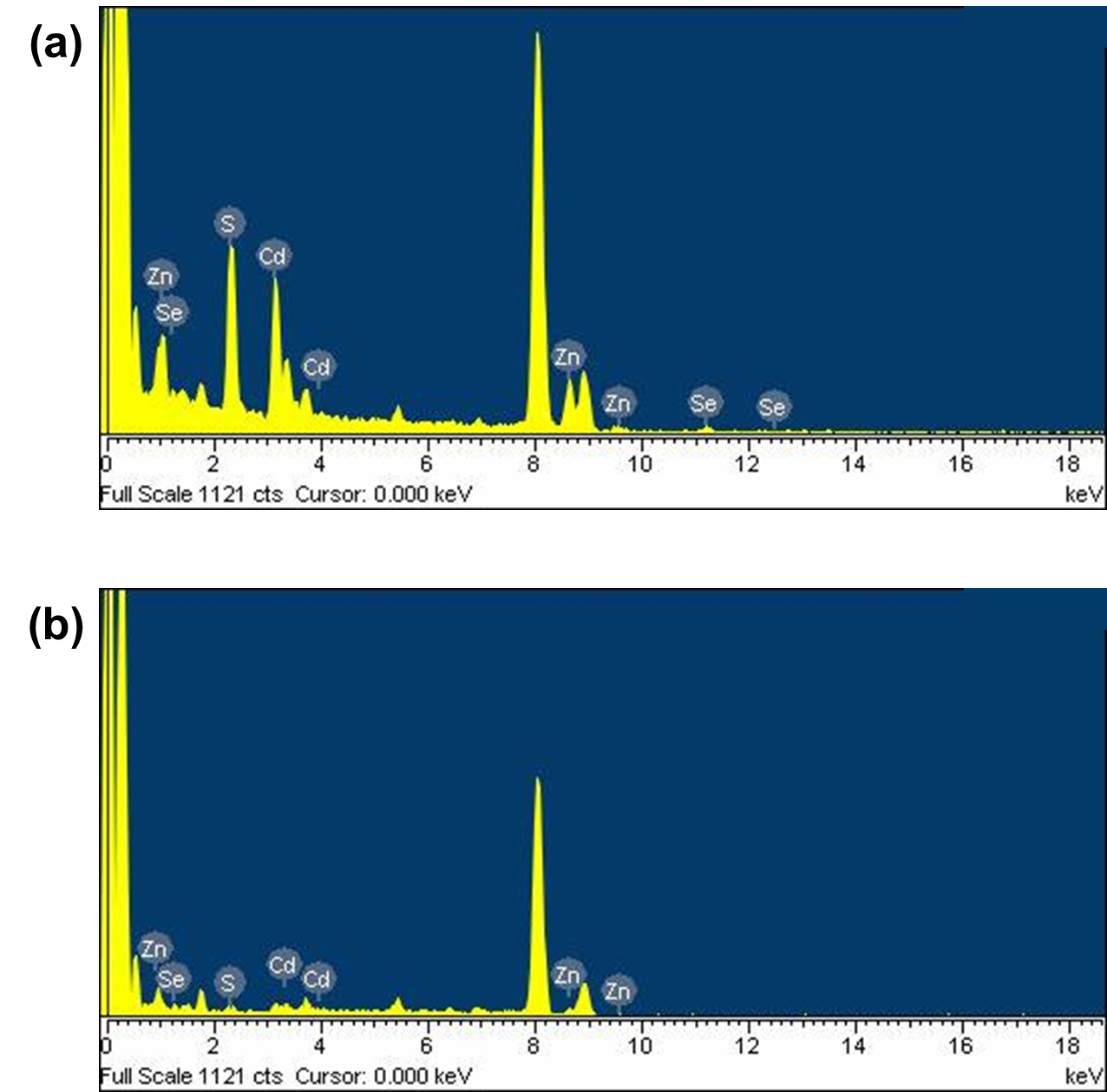
**

**Fig. S1.** EDX spectrum of (a) MQDs and (b) alloy QDs.


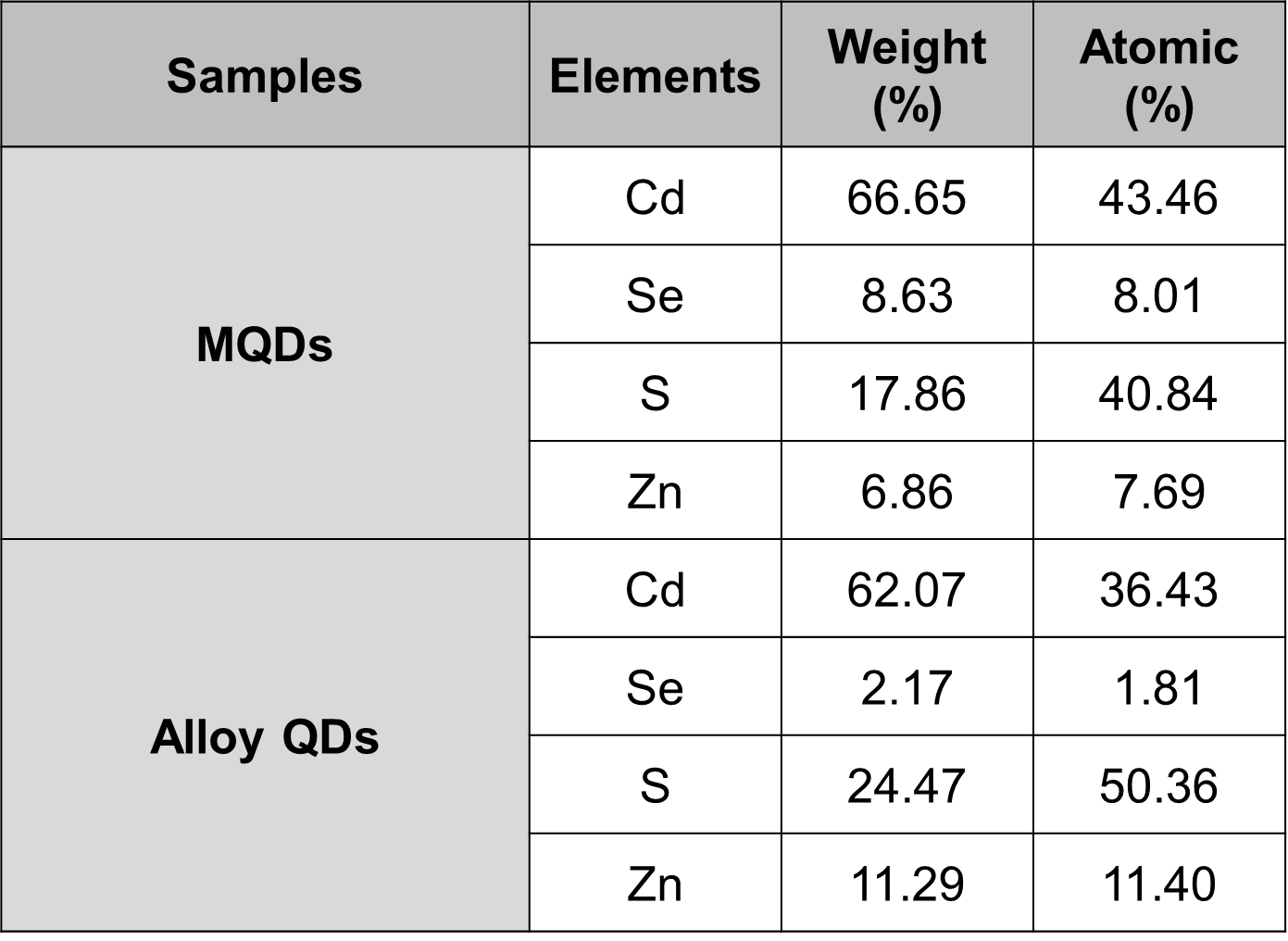


**Table S1.** Atomic composition corresponding to the EDX spectrum of MQDs and alloy QDs.


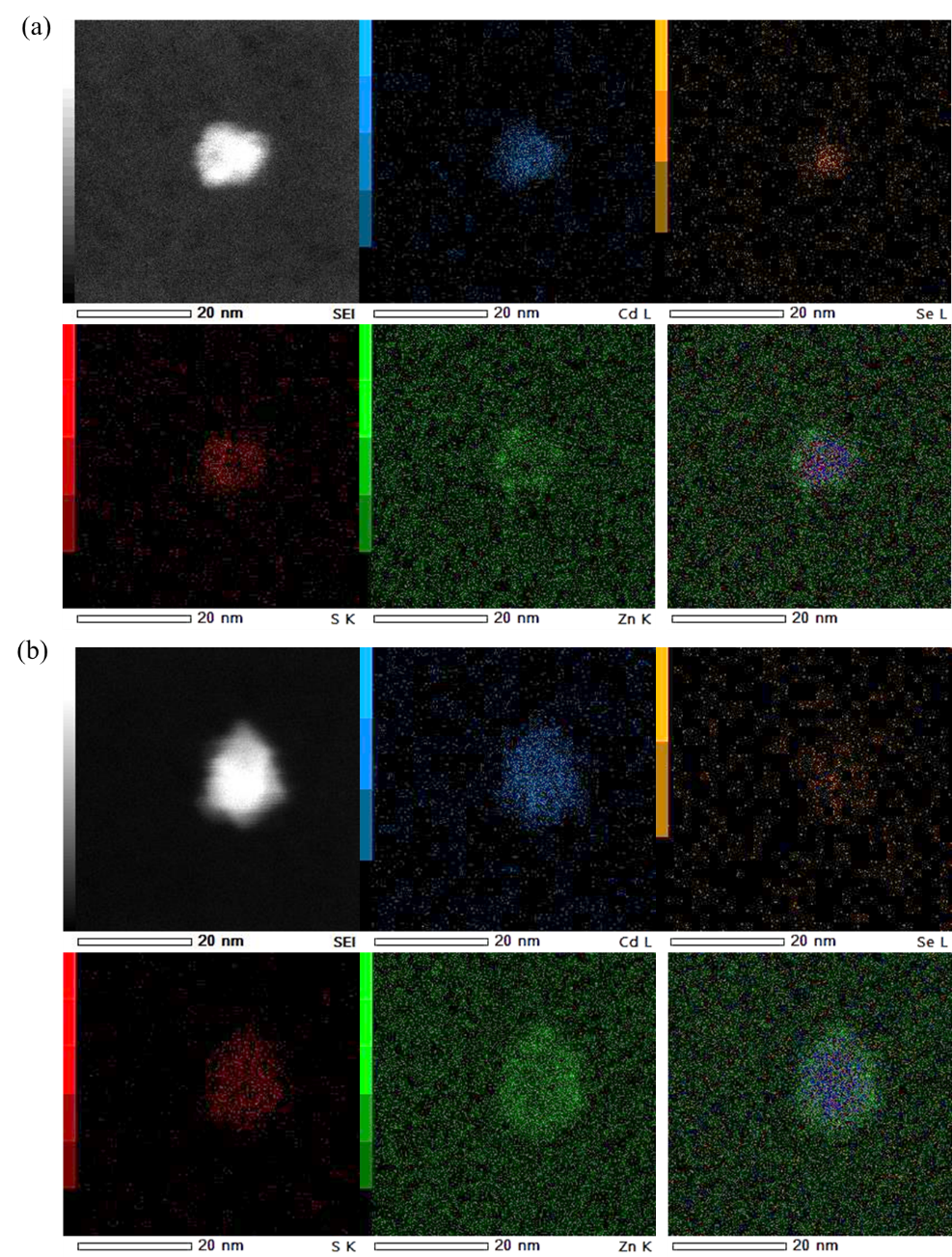


**Fig. S2.** TEM-EDX images of (a) MQDs and (b) Alloy QDs. White colors represented secondary electron imaging (SEI). Blue colors represented Cd. Orange colors represented Se. Red colors represented S. Green colors represented Zn. The images which located at below right of each figure were merged images (Cd, Se, S, and Zn).


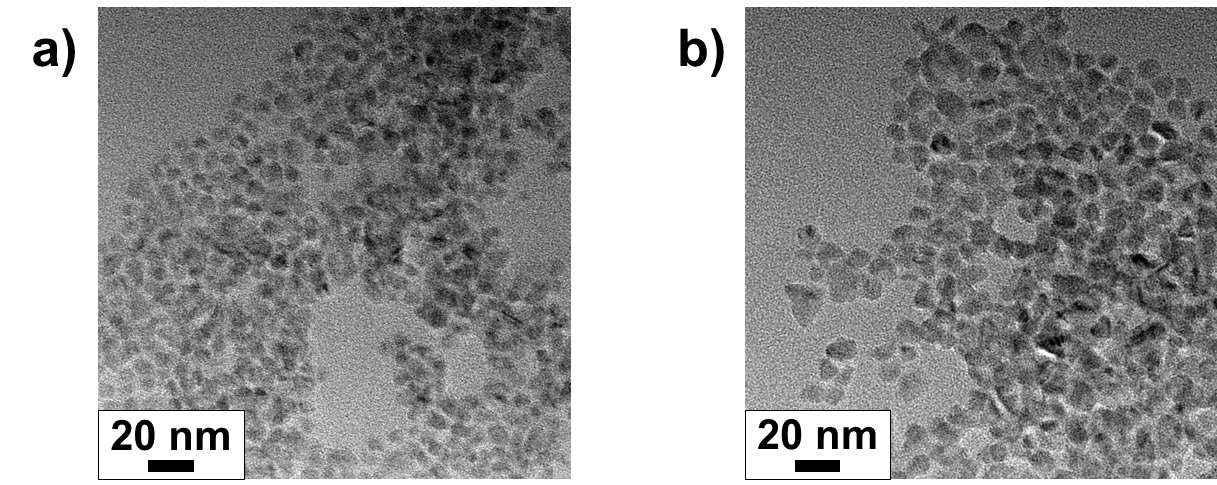


**Fig. S3.** TEM images of a) MQDs-FA and b) alloy QDs-FA


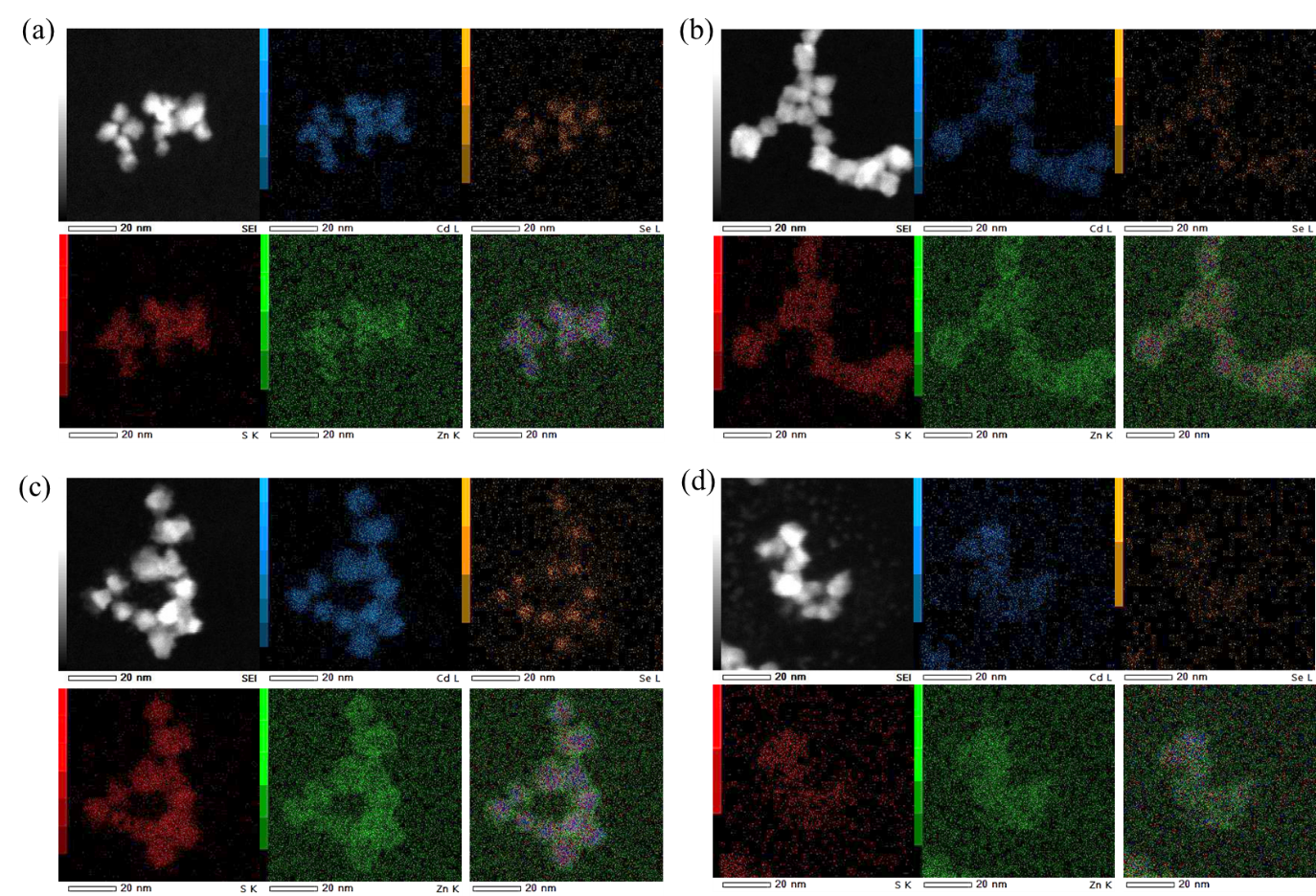


**Fig. S4.** TEM-EDX images of (a) MQDs, (b) Alloy QDs, (c) MQDs-FA, and (d) Alloy QDs-FA. White colors represented secondary electron imaging (SEI). Blue colors represented Cd. Orange colors represented Se. Red colors represented S. Green colors represented Zn. The images which located at below right of each figure were merged images (Cd, Se, S, and Zn).

**Fig. S5.** QY of MQDs and alloy QDs after surface modification (N_3_: 3-Azido-1-propanamine conjugated QDs, DOPA: Dopamine conjugated QDs).

**Fig. S6.** QY of Qdot™ 625 ITK™ Carboxyl Quantum Dots in Invitrogen™ before and after conjugation of FA.


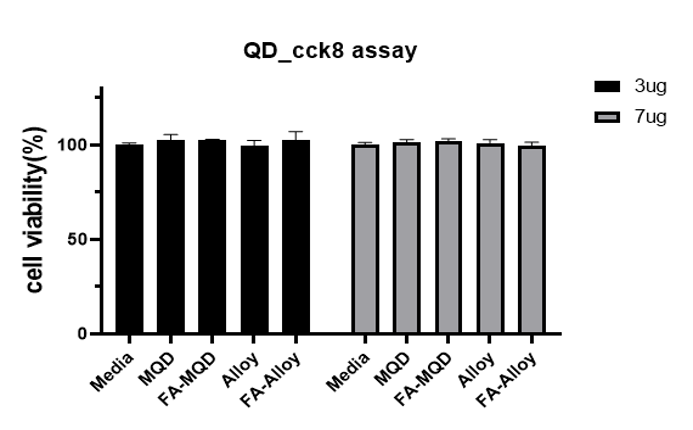


**Fig. S7.** Cell viability test of fabricated QDs via CCK-8 assay.

|  | **PBS** | **MQD** | **Alloy QD** |  | **PBS** | **MQD** | **Alloy QD** |
| --- | --- | --- | --- | --- | --- | --- | --- |
| **BUN** | 27.1 | 26.6 | 27.7 | **CREA** | 0.35 | 0.29 | 0.32 |
| **TP** | 4.9 | 4.5 | 4.7 | **ALB** | 1.8 | 1.7 | 1.8 |
| **T-BIL** | -0.05 | 0.01 | -0.03 | **ALP** | 443 | 413 | 367 |
| **AST (GOT)** | 160 | 83 | 131 | **ALT (GPT)** | 31 | 36 | 54 |
| **GGT** | -1 | 0 | -1 | **CPK** | 983 | 248 | 129 |
| **LDH** | 624 | 259 | 280 | **CRP** | 0.00 | 0.00 | 0.00 |
| **A/G** | 0.6 | 0.6 | 0.6 | **B/C** | 77.43 | 91.72 | 86.56 |

**Table S2.** Protein levels of mice after 24 hours from injection of PBS or QDs. Total protein (TP), blood urea nitrogen (BUN), creatinine (CREA), creatinine phosphokinase (CPK), albumin (ALB), total bilirubin (T-BIL), C-reactive protein (CRP), aspartate aminotransferase (AST), alanine amino Transferase (ALT), γ-glutamyl transferase (GGT), lactate dehydrogenase (LDH), and alkaline phosphatase (ALP) were measured using an automatic analyzer.


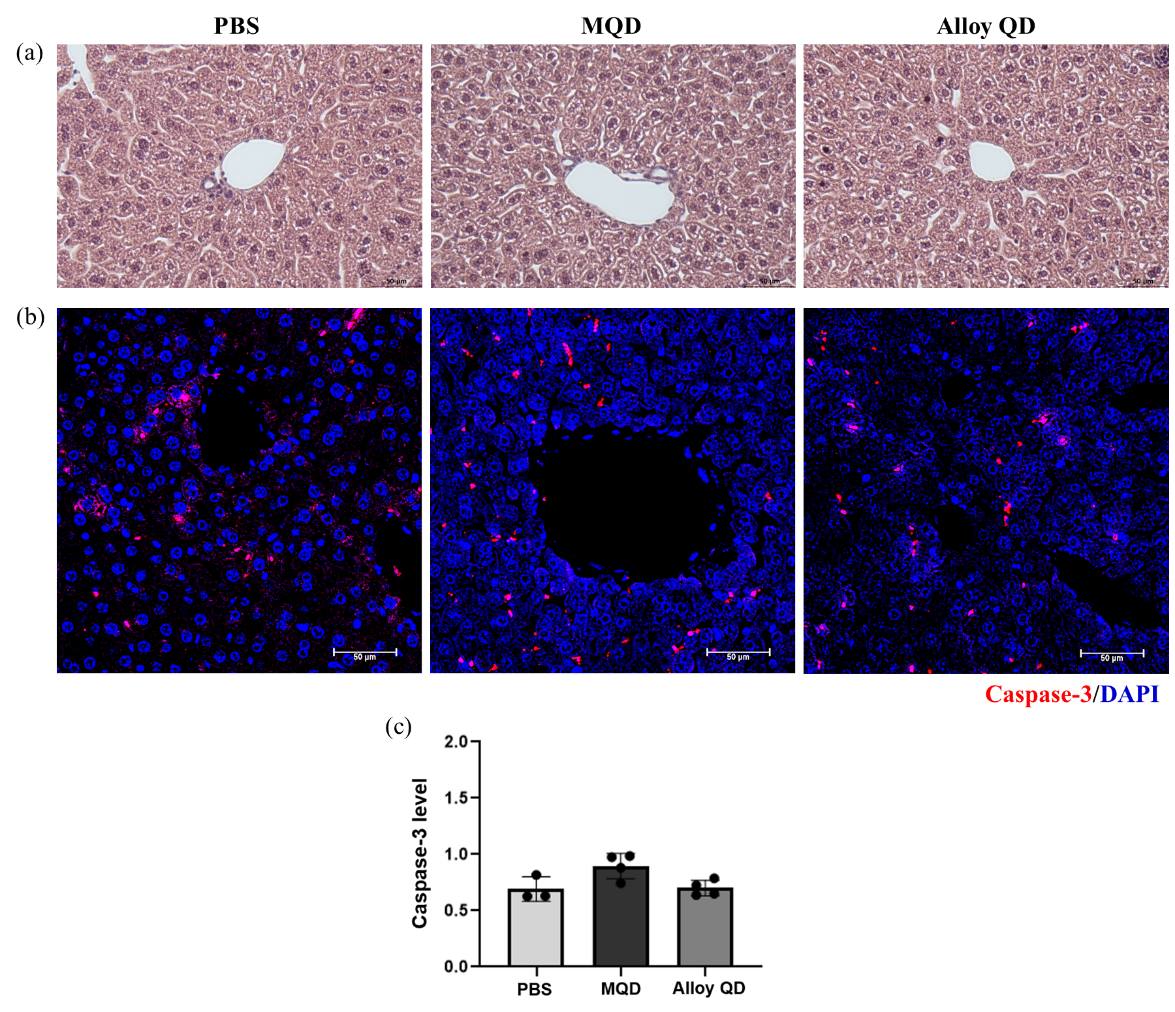


**Fig. S8.** (a) Liver hematoxylin and eosin (H&E) staining of PBS (left), MQD (center), and alloy QD (right) treated mice. (b) Immunohistochemistry staining for caspase-3 detection of PBS (left), MQD (center), and alloy QD (right) treated mice. Cells were stained with DAPI (blue color), and caspase-3 was stained as red color. (c) Caspase-3 level of PBS, MQD, and alloy QD treated mice.
